# A 3D spatial spectral integral equation method for electromagnetic scattering from finite objects in a layered medium

**DOI:** 10.1007/s11082-018-1471-7

**Published:** 2018-04-19

**Authors:** Roeland J. Dilz, Mark G. M. M. van Kraaij, Martijn C. van Beurden

**Affiliations:** 1 Eindhoven University of Technology/EE, PO box 513, 5600 MB Eindhoven, The Netherlands; 2 ASML, De Run 6501, 5504 DR Veldhoven, The Netherlands

**Keywords:** Integral equations, Spectral methods, Gabor frames, Electromagnetic scattering

## Abstract

The generalization of a two-dimensional spatial spectral volume integral equation to a three-dimensional spatial spectral integral equation formulation for electromagnetic scattering from dielectric objects in a stratified dielectric medium is explained. In the spectral domain, the Green function, contrast current density, and scattered electric field are represented on a complex integration manifold that evades the poles and branch cuts that are present in the Green function. In the spatial domain, the field-material interactions are reformulated by a normal-vector field approach, which obeys the Li factorization rules. Numerical evidence is shown that the computation time of this method scales as $O(N \log N)$ on the number of unknowns. The accuracy of the method for three numerical examples is compared to a finite element method reference.

## Introduction

Efficient solvers for electromagnetic scattering in stratified media are important in e.g. metrology (Raymond 2001, Chapter 18), metamaterials (Nanfang et al. 2011; Jahani and Jacob 2016), and integrated optics (Wang et al. 2012). Especially for three-dimensional structures, where the number of unknowns is often very large, there is a demand for fast solvers, for which the computational complexity scales well for large numbers of unknowns. A good strategy to find a potentially efficient algorithm is to exploit symmetries. For stratified media such a symmetry is the translation symmetry in the layered background medium. This symmetry can be exploited through the use of a Fourier representation in a volume integral formulation.

In a stratified dielectric medium, an analytic expression exists for the Green function in the electromagnetic case, as a function of one spatial coordinate in the direction of stratification and two spectral coordinates in the two directions perpendicular to the stratification (Kong 1975; Felsen and Marcuvitz 1973; Chew 1995; Michalski and Mosig 1997). It is advantageous to use the stratified-medium Green function, since it incorporates the response of the multilayer medium analytically. Therefore, little computation time or memory is used for computing the scattered electromagnetic field throughout the entire layered stack, since the electric field on a domain slightly larger than the scattering object suffices. It is possible, using Sommerfeld (1909) or Fourier integrals, to transform the Green function completely to the spatial domain and then use it in an integral equation method (Chew 1995, Chapter 8; Felsen and Marcuvitz 1973, Chapter 5; Kong 1975, Chapter 4; Wait 1970, Chapter 2). However, these Sommerfeld integrals are often tedious to compute, because of poles and branch cuts present in the Green function that can be located on or close to the integration path. Since the Green function has to be re-calculated for every modification in the multilayer medium, caching the Green function in a library is only advantageous when exactly the same multilayered medium is used many times.

It is also possible to use the Green function directly in the spectral domain, where it is known analytically. For a periodically repeating object, the Green function decomposes into a discrete set of modes as derived in for example (Beurden 2011, 2012). Problems with poles and oscillations along branch cuts in the Green function (Chew 1995; Felsen and Marcuvitz 1973) can be avoided on such a discrete set of modes since the modes and locations of the poles will most likely not coincide. However, for a finite scatterer the spectral domain is continuous and now the poles and oscillations along the branch cuts are hard to discretize (Dilz and Beurden 2016, 2017). Deformations of the Sommerfeld integration path to a complex-plane path (Ruiter 1981; Newman and Forrai 1987; Hochman and Leviatan 2010; Michalski and Mosig 2016) can help to evade these poles and branch cuts. In Dilz and Beurden (2017) an algorithm for two-dimensional electromagnetic scattering with TE polarization in a multilayered medium is presented, where both contrast-current density and scattered field are represented on a path in the complex plane of the spectral domain. It is this path that allows for the use of Gohberg and Koltracht (1985) fast, flexible and recursive Green-function convolution in the stratification direction.

The first challenge in three dimensions is that, instead of one, now two directions perpendicular to the stratification direction need to be handled. The complex integration path is turned into a complex integration manifold and since the transformation from the spatial domain to the complex integration manifold is part of the core of the algorithm, transformations back and forth need to be computationally efficient. We show an integration plane consisting of nine regions of three distinct types and show transformations to and from the spatial domain that can be computed in $O(N \log N)$ time, where *N* is the number of spectral unknowns.

The second challenge is that the discontinuity of both the permittivity and the electric field at material interfaces leads to poor convergence in spectral formulations (Li and Haggans 1993). This effect was also observed for a Gabor-frame based solver for TM-polarized scattering (Dilz et al. 2017). For periodic scattering problems with a discrete spectral expansion a reformulation of the field-material interactions corrects this poor rate of convergence (Granet and Guizal 1996; Lalanne and Morris 1996), which is explained in more detail in Li (1996) introducing the so-called Li-rules. In Dilz et al. (2017) it is shown that the same mechanism can also be used for a continuous spectral expansion and the algorithm of Dilz and Beurden (2017) is extended to efficiently deal with the discontinuous field-material interaction in a way that does abide these Li-rules. Here, we propose a generalization of this method to three dimensions. Inspired by Beurden and Setija (2017), we show that a normal-vector field formulation (Popov and Nevire 2001) can be used for three-dimensional scattering to replace the field-material interaction.

We start by a short formulation of the volume integral equation. Subsequently, we give a more detailed explanation of the discretization, with emphasis on the complex-plane spectral domain representation, followed by a short summary of the normal-vector field framework. The applicability of the present algorithm is highlighted by three numerical examples, with numerical evidence that the computation time scales as $O(N \log N)$ with the number of unknowns and comparison against a finite-element reference calculation.

## The volume integral equation

**Fig. 1:**
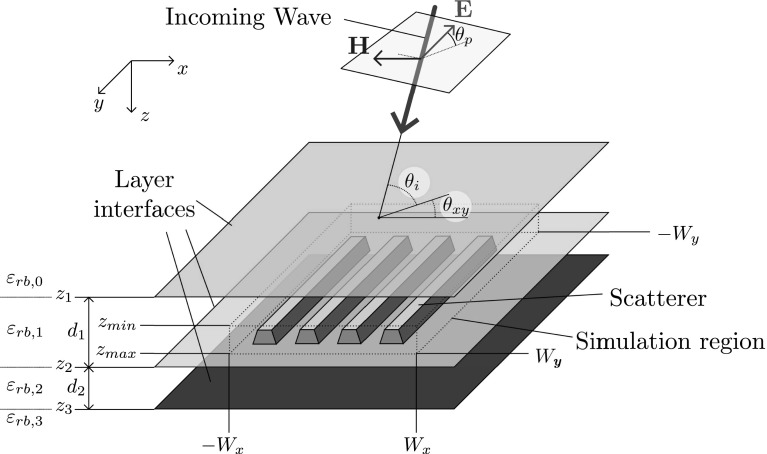
An illustration of a possible scattering setup

Consider a stratified dielectric medium where layers with different relative permittivities are stacked in the *z*-direction. Layer *n* is located between $z_n$ and $z_{n+1}$ and has relative permittivity $\varepsilon _{rb,n}$. Index $n=0$ coincides with the top half-space, $z<0$, and index $n=N_L$ with the half-space $z>z_{N_L+1}$ below all layers, an example of which is also illustrated in Fig. 1. In layer *i* a three-dimensional dielectric object is contained within the simulation domain ${\mathscr {D}} = [-W_x,W_x]\times [-W_y,W_y] \times [z_{min},z_{max}]$, with $z_{i}\le z_{min}$ and $z_{max} \le z_{i+1}$. This dielectric object is characterised by a relative permittivity function $\varepsilon _r(\mathbf{x})$, with $\mathbf{x}=(x,y,z)$, or more conveniently by the contrast function

1 $$\begin{aligned} \chi (\mathbf{x}) = \frac{\varepsilon _r(\mathbf{x})}{\varepsilon _{rb,i}}-1, \end{aligned}$$

which is nonzero only in the object.

An incident electromagnetic field originates from the upper half-space at arbitrary angles and polarization. The electric field in presence of the multilayered background medium $\varepsilon _{rb,n}$, but in absence of the dielectric object can be readily calculated (Chew 1995; van Kraaij 2011) and is denoted as $\mathbf{E}^i(\mathbf{x})$. The dielectric object generates a scattered field $\mathbf{E}^s(\mathbf{x})$ that together with the incident field $\mathbf{E}^i(\mathbf{x})$ adds up to the total electric field $\mathbf{E}(\mathbf{x})$, i.e.

2 $$\begin{aligned} \mathbf{E}(\mathbf{x}) = \mathbf{E}^i(\mathbf{x}) + \mathbf{E}^s(\mathbf{x}). \end{aligned}$$

The scattered field $\mathbf{E}^s(\mathbf{x})$ can be calculated via the multilayer Green tensor through

3 $$\begin{aligned} \mathbf{E}^s(\mathbf{x}) = \int _{{\mathscr {D}}}^{} d \mathbf{x}' \, {\mathscr {G}}(\mathbf{x}|\mathbf{x}') \cdot \mathbf{J}(\mathbf{x}'), \end{aligned}$$

where the contrast current density $\mathbf{J}(\mathbf{x})$ is given by the field-material interaction

4 $$\begin{aligned} \mathbf{J}(\mathbf{x}) = j \omega \varepsilon _0 \varepsilon _{rb,i} \chi (\mathbf{x}) \mathbf{E}(\mathbf{x}), \end{aligned}$$

which is again nonzero only in the scattering object. Combining Eqs. (2)–(4) yields the integral equation that we propose to solve

5 $$\begin{aligned} \mathbf{E}^i(\mathbf{x}) = \mathbf{E}(\mathbf{x}) - \int _{{\mathscr {D}}}^{} d \mathbf{x}' \, {\mathscr {G}}(\mathbf{x}|\mathbf{x}') \cdot \left[ j \omega \varepsilon _0 \varepsilon _{rb,i} \chi (\mathbf{x}') \mathbf{E}(\mathbf{x}') \right] . \end{aligned}$$

However, for an efficient numerical scheme several refinements have to be made.

## The spectral domain representation

### The Green function

The three-dimensional integral in Eq. (3) yields, when implemented naively, an $O(N^2)$ matrix-vector product, with *N* the total number of unknowns and by employing an iterative solver. Analoguous to Dilz and Beurden (2016, 2017), Dilz et al. (2017), we represent the Green function, the contrast current density $\mathbf{J}$, and scattered field $\mathbf{E}^s$ in the spectral domain in the transverse *xy*-plane. We denote coordinates in the transverse plane as $\mathbf{x}_T= (x,y)$, and in the spectral domain as $\mathbf{k}_T = (k_x,k_y)$. We use a Fourier transformation defined as

6 $$\begin{aligned} f(k_\xi ) = {\mathscr {F}}_\xi \left[ f(\xi ) \right] (k_\xi ) = \int _{-\infty }^{\infty }d\xi \, f(\xi ) e^{-j k_\xi \xi }, \end{aligned}$$

where we distinguish functions in the spectral domain by arguments containing $k_x$, $k_y$ and $\mathbf{k}_T$ and in the spatial domain by the arguments *x* and *y* and $\mathbf{x}_T$.

In the spectral domain, a spatial convolution can be executed with $O(N_x N_y)$ complexity, with $N_\alpha$ the number of unknowns used in direction $\alpha$. The transverse convolution in Eq. (5) can be carried out efficiently. The remaining integration in the *z*-direction can be calculated in $O(N_z)$ time via the recursive algorithm proposed by Gohberg and Koltracht (1985).

The multilayer Green tensor in Eq. (3), can be separated in a homogeneous-medium part yielding $\mathbf{E}^{s,h}$ and reflected waves moving up, $\mathbf{K}^u$, and down, $\mathbf{K}^d$. The homogeneous-medium part of the scattered field is given by

7 $$\begin{aligned} \mathbf{E}^{s,h}(\mathbf{k}_T,z) = \int _{z_{min}}^{z_{max}}dz' \, {\mathscr {G}}^h(\mathbf{k}_T,z|z') \cdot \mathbf{J}(\mathbf{k}_T,z'), \end{aligned}$$

where the homogeneous-medium Green tensor is given in Cartesian components (*x*, *y*, *z*), respectively, as

8 $$\begin{aligned} {\mathscr {G}}^h(k_x,k_y,z|z') = \left( \begin{array}{ccc} \varepsilon _{rb,i} k_0^2 - k_x^2 &{}\quad - k_x k_y &{}\quad - k_x \gamma \\ - k_x k_y &{}\quad \varepsilon _{rb,i}k_0^2 - k_y^2 &{}\quad - k_y \gamma \\ - k_x \gamma &{}\quad - k_y \gamma &{}\quad \gamma ^2 -\frac{2 \delta (z-z')}{\gamma } \end{array} \right) \frac{e^{-\gamma |z-z'|}}{2 \gamma }. \end{aligned}$$

Here, $k_0$ is the wave number $k_0 =\omega \sqrt{ \mu _0 \varepsilon _0}$ and $\gamma$ is defined as $\gamma = \sqrt{\mathbf{k}_T^2-\varepsilon _{rb,i} k_0^2}$, where $\mathbf{k}_T = (k_x,k_y,0)$ so $\mathbf{k}_T^2=k_x^2+k_y^2$. Note that the factor $\exp (-\gamma |z-z'|)$ propagates the electric field over a distance $|z-z'|$, and will therefore be referred to as the propagation function.

Now the scattered field $\mathbf{E}^s$ can found by adding reflected waves $\mathbf{K}^{u/d}$ from the layer interfaces to the homogeneous scattered field $\mathbf{E}^{s,h}$, where *u* and *d* refer to waves moving up or down respectively. Consequently, we have

9 $$\begin{aligned} \begin{aligned} \mathbf{E}^s(\mathbf{k}_T,z) =&\mathbf{E}^{s,h}(\mathbf{k}_T,z) \\&+\left( {\mathscr {R}}^{u,u}(\mathbf{k}_T,z) \mathbf{E}^{s,h}(\mathbf{k}_T,z_{min}) + {\mathscr {R}}^{u,d}(\mathbf{k}_T,z) \mathbf{E}^{s,h}(\mathbf{k}_T,z_{max}) \right) e^{-\gamma (z-z_{min})} \\&+ \left( {\mathscr {R}}^{d,u}(\mathbf{k}_T,z) \mathbf{E}^{s,h}(\mathbf{k}_T,z_{min}) + {\mathscr {R}}^{d,d}(\mathbf{k}_T,z) \mathbf{E}^{s,h}(\mathbf{k}_T,z_{max}) \right) e^{-\gamma (z_{max}-z)} \end{aligned} \end{aligned}$$

with ${\mathscr {R}}^{\alpha ,\beta }(\mathbf{k}_T,z)$ the three-dimensional effective reflection coefficient that contains both *h* and *e* polarization, which can be calculated from the effective reflection coefficients for *h* polarization $r^{\alpha ,\beta }_h(\mathbf{k}_T)$ (Dilz and Beurden 2017), and for *e* polarization $r^{\alpha ,\beta }_e(\mathbf{k}_T)$ (Dilz et al. 2017) as

10 $$\begin{aligned} {\mathscr {R}}^{\alpha ,\beta } = \left( \begin{array}{ccc} \frac{k_x^2 r_e^{\alpha ,\beta }(\mathbf{k}_T) - k_y^2 r_h^{\alpha ,\beta }(\mathbf{k}_T)}{\mathbf{k}_T^2} &{} \frac{k_x k_y (r_e^{\alpha ,\beta }(\mathbf{k}_T)- r_h^{\alpha ,\beta }(\mathbf{k}_T))}{\mathbf{k}_T^2} &{} 0\\ \frac{k_y k_x (r_e^{\alpha ,\beta } (\mathbf{k}_T)-r_h^{\alpha ,\beta }(\mathbf{k}_T))}{\mathbf{k}_T^2} &{} \frac{k_y^2 r_e^{\alpha ,\beta }(\mathbf{k}_T) - k_x^2 r_h^{\alpha ,\beta }(\mathbf{k}_T)}{\mathbf{k}_T^2} &{} 0\\ 0 &{} 0 &{} r_e^{\alpha ,\beta } (\mathbf{k}_T) \end{array} \right) . \end{aligned}$$

This matrix projects the *e* and *h* polarized parts of the electric field to effective transmission coefficients $r_e^{\alpha ,\beta }$ and $r_h^{\alpha , \beta }$, respectively. The definition of these effective reflection coefficients is given in Dilz and Beurden (2017), Dilz et al. (2017), which is based on the expositions about multilayer media in Kong (1975, Chapter 4), (Wait 1970, Chapter 2), van Kraaij (2011).

Since the field-material interaction in Eq. (4) is calculated in the spatial domain and the Green-function operation in Eq. (9) in the spectral domain, we need a fast and efficient means of transforming the current density $\mathbf{J}(\mathbf{x}_T,z)$ to the spectral domain and the scattered field $\mathbf{E}^s(\mathbf{k}_T,z)$ back to the spatial domain. We propose to use a two-dimensional Gabor-frame in the transverse plane, since a Gabor frame is efficient to represent the operation of Fourier transformation. It can be represented analytically by a mere transposition of the coefficient matrix in *O*(*N*) operations (Dilz and Beurden 2016).

### The Gabor frame

We use a Gabor frame with Gaussian window function

11 $$\begin{aligned} g(x,y) = 2^{\frac{1}{2}}\exp \left( - \pi \frac{x^2}{X^2} - \pi \frac{y^2}{Y^2}\right) , \end{aligned}$$

with width *X* in the *x*-direction and *Y* in the *y*-direction. This defines the oversampled two-dimensional Gabor frame as

12 $$\begin{aligned} g_{\mathbf{m}\mathbf{n}}(x)= g(x-m_x \alpha X,y-m_y \alpha Y) e^{j n_x \beta K_x x + j n_y \beta K_y y}, \end{aligned}$$

with two-dimensional indices $\mathbf{m}=(m_x,m_y)$ and $\mathbf{n}=(n_x,n_y)$. Here, the spectral spacing is $K_x = 2\pi /X$ and $K_y = 2 \pi /Y$ and oversampling $\alpha _x \beta _x < 1$ and $\alpha _y \beta _y <1$. The number of coefficients in $\mathbf{m}$ and $\mathbf{n}$ is allowed for to be different in the two directions. Gabor coefficients can be calculated as

13 $$\begin{aligned} f_{\mathbf{m}\mathbf{n}} = \int _{-\infty }^{\infty }dx\int _{-\infty }^{\infty }dy \, f(x,y) \eta _{\mathbf{m}\mathbf{n}}(x,y), \end{aligned}$$

with dual frame

14 $$\begin{aligned} \eta _{\mathbf{m}\mathbf{n}}(x,y)= \eta (x-m_x \alpha X)\eta (y-m_y \alpha Y) e^{j n_x \beta K_x x + j n_y \beta K_y y}. \end{aligned}$$

There is freedom of choice for the dual window function $\eta (x)$, but we choose the dual frame function calculated via the Moore Penrose inverse (Feichtinger and Strohmer 1998; Bastiaans 1995), since it is widely used and exhibits a convenient exponential decay in both the spatial and spectral domain.

We use the Fourier transformation of Eq. (12) to discretize functions in the spectral domain. This has the advantage that the operation of Fourier transformation reduces to merely a tranposition of coefficients. Details on operations such as Fourier transformation and multiplication of Gabor-represented functions can be found in Dilz and Beurden (2016) for one dimension and the generalization to two dimensions is straightforward.

## A complex-plane deformation of the integration manifold

In the *z*-direction, the integration with the Green tensor in Eq. (7) is discretized completely in the spatial domain. Since it was shown that a piecewise-linear approximation in the *z*-direction is effective (Dilz and Beurden 2016, 2017; Dilz et al. 2017), we propose to use it here again. In the *z*-direction, the basis functions are then defined as

15 $$\begin{aligned} \varLambda _\ell (z) = {\left\{ \begin{array}{ll} 1-\frac{|z - \ell \varDelta _z-z_{min}|}{ \varDelta _z} &{}\quad \text {if} \quad |z-\ell \varDelta _z-z_{min}|<\varDelta _z \\ 0 &{}\quad \text {if} \quad |z-\ell \varDelta _z-z_{min}|>\varDelta _z \end{array}\right. }, \end{aligned}$$

with $\varDelta _z$ the discretization step in the *z*-direction.

For the discretization in the *xy* plane, a method similar to the two-dimensional cases in Dilz and Beurden (2017), Dilz et al. (2017) is proposed. The Green function contains poles due to the effective reflection coefficients and many oscillations along the branch cuts may occur. Both these poles and oscillations cannot be represented efficiently in a Gabor frame representation. In the two-dimensional case, these problems can be circumvented by representing the Green-function in the transverse direction in Eq. (9) on a path in the spectral complex plane. For three-dimensional problems, this path can be generalized to a two-dimensional integration manifold in the transverse $\mathbf{k}_T$ coordinates on which the transformation back to the spatial domain takes place. In the $k_x$-direction, the complex spectral path is defined by the function $\tau _x(k_x)$, with $k_x \in \mathbb {R}$ and $\tau _x \in \mathbb {C}$ as

16 $$\begin{aligned} \tau _x(k_x) \in {\left\{ \begin{array}{ll} k_x-j A_x &{}\quad \text {if } k_x< -A_x \\ (1+j) k_x &{}\quad \text {if } -A_x \le k_x < A_x \\ k_x + j A_x &{}\quad \text {if } k_x > A_x. \end{array}\right. } \end{aligned}$$

and similarly for $\tau _y(k_y)$ with $A_y$ defining the path along the $k_y$-direction. Here, $A_x$ and $A_y$ are constants that can be chosen individually. Numerical experiments show that a choice such that $A_x W_x$ and $A_y W_y$ are in the range $2 \dots 5$, yields optimal accuracy, with $W_x$ and $W_y$ as in Fig. 1. With the coordinate change from $\mathbf{k}_T$ to $\mathbf{\tau }_T$, Eqs. (7) and (9) contain smooth functions and these can be used in combination with the Gabor-frame discretization.

**Fig. 2:**
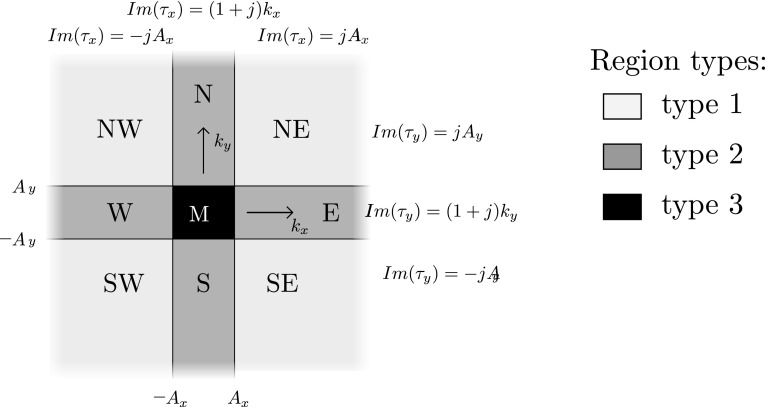
The complex-plane integration domain in the spectral domain consisting of nine regions, of three types

This complex spectral manifold divides the complex $\mathbf{k}_T$ domain into nine regions as depicted in Fig. 2. All functions in the spectral domain will be represented on this $\tau _T$ manifold. Using Jordan’s lemma, the Fourier transformation to the spatial domain can be carried out over the $\tau _T$ manifold. Closing the contour at $\mathbf{k}_T \rightarrow \infty$ is not needed, since the representation using Gabor frames converges to zero rapidly.

It should be noted that the complex integration path of (16) is not the only possible choice. For example, in Dilz and Beurden (2018) a different continuous path in one dimension is chosen. The current choice for the path in (16) was made because the large horizontal stretches allow for fast transformations to and from the spectral domain. Different choices might not allow for such computational efficiency.

### Discretization in regions of type 1

Most information is contained in regions of Type 1, since $A_x$ and $A_y$ are relatively small compared to the complete spectral range to be discretized. The contrast current density is transformed to the complex spectral integration manifold via

17 $$\begin{aligned} \mathbf{J}(k_x+j A_x,k_y+j A_y,z) = {\mathscr {F}}_{\mathbf{x}_T}[\mathbf{J}(\mathbf{x}_T,z) e^{x A_x + y A_y}](\mathbf{k}_T), \end{aligned}$$

for the northeast (NE) quadrant, i.e. $k_x \ge A_x \wedge k_y \ge A_y$, and similarly for the other regions of Type 1. Analogously, the transformation of the scattered field back to the spatial domain is obtained as

18 $$\begin{aligned} \mathbf{E}^{s,\text {NE}}(\mathbf{x}_T,z) = e^{-x A_x - y A_y}{\mathscr {F}}^{-1}_{\mathbf{k}_T}[c_\text {NE}(\mathbf{k}_T) \mathbf{E}^{s,\text {NE}}(k_x+j A_x,k_y+j A_y,z)](\mathbf{x}_T), \end{aligned}$$

with the cut-off function $c_{\text {NE}}(\mathbf{k}_T)$ equalling 1 on the NE region and zero elsewhere. The Fourier transformation can be performed in *O*(*N*) operations and the operation of multipication in $O(N \log N)$ operations, for functions represented by *N* Gabor coefficients. Therefore, the total of these operations allows for an $O(N \log N)$ computational complexity.

All this means that the scattered electric field $\mathbf{E}^s$ is represented by a five-dimensional array of coefficients $\mathbf{E}^{s,\text {NE}}_{\mathbf{m},\mathbf{n},l}$, with $m_x,n_x$ and $m_y,n_y$ corresponding to the Gabor frame on the coordinates, $k_x+j A_x$ and $k_y+j A_y$ respectively. The $\ell$ index corresponds to a piecewise-linear (PWL) representation in the *z*-direction. The scattered electric field in region NE is then approximated as

19 $$\begin{aligned} \mathbf{E}^{s,\text {NE}}(k_x+j A_x,k_y+j A_y,z) \approx \sum _{\mathbf{m},\mathbf{n}}^{} \sum _{\ell =1}^{N_z} g_{\mathbf{m},\mathbf{n}}(k_x,k_y) \varLambda _\ell (z) \mathbf{E}^s_{\mathbf{m},\mathbf{n},\ell }. \end{aligned}$$

**Fig. 3:**
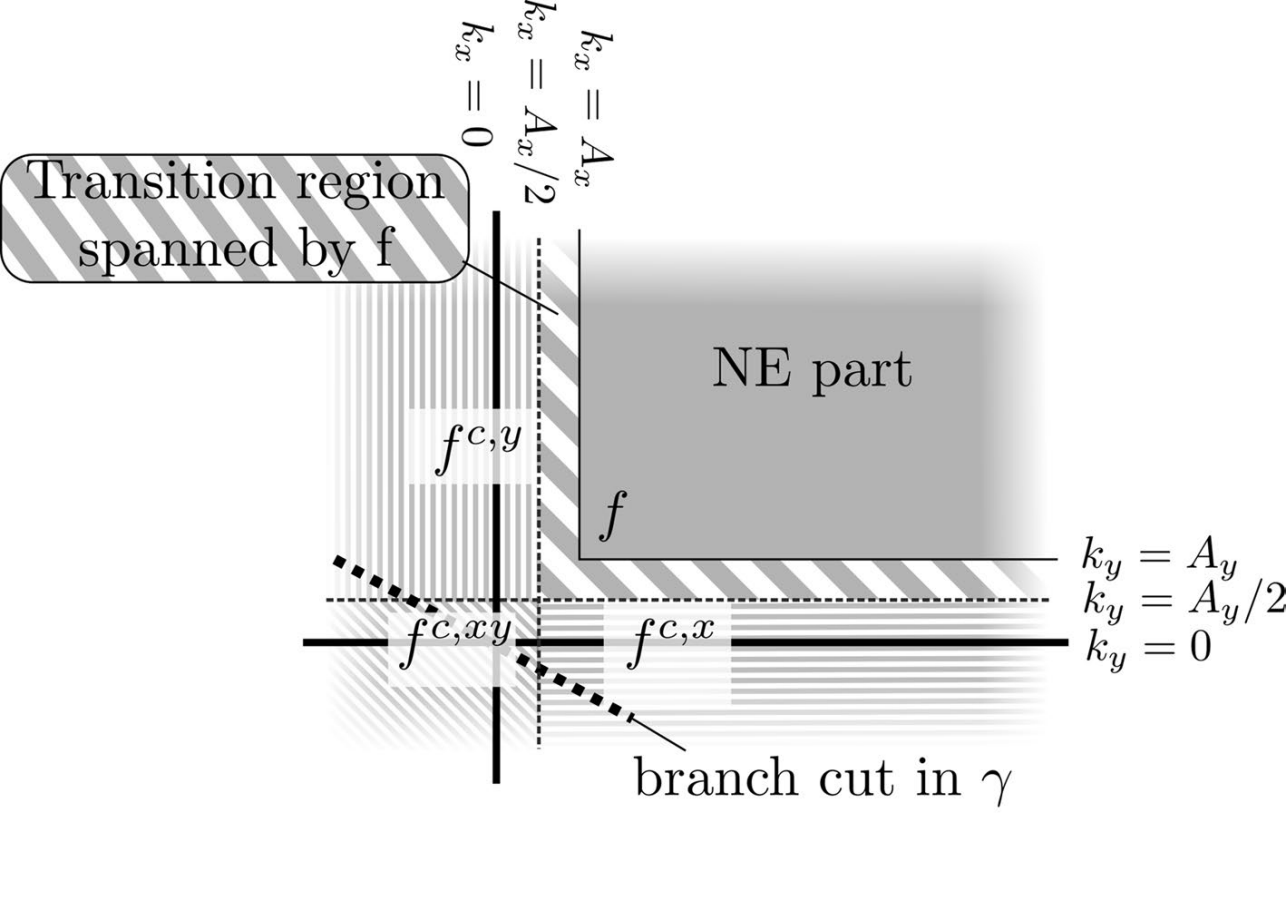
A function *f* represented in the NE regions on $\tau (k_x,k_y) =(k_x+jA_x,k_y+jA_y)$ that depends on $\gamma (\tau (\mathbf{k}_T))$ contains a branchcut on a straight line through the $k_x<0 \vee k_y<0$ region. On the regions indicated with solid and striped grey the original function *f* is discretized and on regions indicated by fine lines the continuations $f^{c,x}$, $f^{c,xy}$ and *f* *c*, *y* are discretized, the discontinuity of the branch cut is therefore avoided by the continuous functions

The Green function consists of several parts, some of which are depending on the complex propagation constant $\gamma (\mathbf{k}_T) = \sqrt{-\varepsilon _{rb,i} k_0^2+\mathbf{k}_T^2}$. On the real $k_x k_y$-plane $\gamma (\mathbf{k}_T)$ touches, but does not cross, two branch cuts at $\mathbf{k}_t = (0,0)$ in the case of lossless media. For lossy media the branchcuts are located at some distance from the origin. For both cases, the $\tau$ path passes just in between these two branchcuts. However, when a Type-1 region such as the NE-region is continued to the complete $(k_x+jA_x, k_y + jA_y)$ plane, a branch cut is crossed just outside the NE region, as illustrated in Fig. 3. The branch cut is located on a straight line through $\tau _T = (0+jA_x,0+j A_y)$ and direction of the line depends on the choice of $A_x$ and $A_y$. The continuous nature of a Gabor-frame representation does not allow for an abrupt stop of the discretization domain at the borders of a Type-1 region. Therefore, such a Gabor-frame representation of the Green function exhibits significant Gibbs ringing from the branch cut that spreads into the Type-1 regions. For a two-dimensional case, this is described in Dilz and Beurden (2017), where a linear continuation of the Green function is proposed that suppresses strong Gibbs ringing.

In three dimensions, this issue can also be resolved by making a first-order continuation of the functions to eliminate the branch cut. Since the branch cut can be located close to the $k_x=0$ or $k_y=0$ axes, and the function values are needed at $k_x>A_x$ and $k_y>A_y$, we start the continuation of the functions in the middle at $k_x^c = A_x/2$ and $k_y^c = A_y/2$. Then the Gibbs phenomenon from the discontinuous second derivative will be at a short distance from $k_x = A_x$ and $k_y = A_y$, where Region NE begins. For the continuation of a function $f(k_x,k_y)$ along the $k_y$-axis we choose

20 $$\begin{aligned} f^{c,x}(\mathbf{k}_T) = \left[ f(\frac{A_x}{2},k_y) + (k_x-\frac{A_x}{2} ) \partial _{k_x} f(\frac{A_x}{2},k_y)\right] e^{-\alpha (k_x-A_x/2)^2}, \end{aligned}$$

for $k_x<A_x/2$ and $k_y>A_y/2$ and similarly $f^{c,y}(k_x,k_y)$ can be constructed for $k_x>A_x/2$ and $k_y<A_y/2$, which is illustrated in Fig. 3. The Gaussian factor is added to make the continuation decay to zero slowly. The third part, $k_x<A_x/2$, $k_y<A_y/2$ is a continuation of $f^{c,y}$ into

21 $$\begin{aligned} f^{c,xy}(\mathbf{k}_T) = \left[ f^{c,y}(\frac{A_x}{2},k_y) + (k_x-\frac{A_x}{2} ) \partial _{k_x} f^{c,y}(\frac{A_x}{2},k_y)\right] e^{-\alpha (k_y-A_y/2)^2}. \end{aligned}$$

Note that this expression equals the expression obtained from continuing $f^{c,x}$ onto this domain. The derivative of the function *f* is calculated using a forward finite-difference method, with a difference of $10^{-4} A_x$ or $10^{-4} A_y$ for the *x* and *y* direction, respectively. For most functions, $\alpha = \min (X^2,Y^2)$ is a good choice. However, for one part of the Green function, notably the propagation function $e^{-\gamma \varDelta _z}$, care has to be taken that its absolute value does not exceed one in the continuation. By increasing the value of $\alpha$, this condition can always be satisfied. More details can be found in Dilz and Beurden (2017).

A general remark about the importance of this continuation is in place. In principle, the Gibbs phenomenon in a Gabor frame representation is not of much significance, unless two functions with discontinuities at the same position are multiplied. The Li-rules (1996) state that when two functions with spatial discontinuities at the same position are multiplied to form a convolution in the spectral domain, the convergence of this convolution is poor. The Li-rules also apply to Gabor frames (Dilz et al. 2017) and since the spatial and spectral domain are both represented by a Gabor frame, a spatial version of the Li-rules is also applicable to the Gabor frame. These spatial Li-rules state that when two spectral functions with discontinuities are multiplied in a Gabor-frame representation, a poor convergence is observed. Now when the NE region of the electric field with its branch cut is multiplied by the cut-off function $c_\text {NE}(\mathbf{k}_T)$ in Eq. (18), which is discontinuous at $k_x = A_x$ and at $k_y = A_y$, functions are multiplied for which the locations of discontinuities almost touch each other. This leads to near-violation of the spatial Li-rules. Since the discontinuities are not exactly at the same location, a high sampling would in principle solve this issue. However, this would require an excessive sample density that is avoided by the continuation of the Green function parts proposed in Eqs. (20) and (21).

### Discretization in regions of type 2

First we will approximate the contrast current density in $k_x$ around $k_x=0$ with a Taylor expansion that is found through a Vandermonde matrix. This Taylor expansion is then applied to find corresponding values of the contrast current density on the line $Im(\tau _x) = Re(\tau _x)$, on which a PWL basis is used as a discretization. This PWL basis consists of $2 N_s+1$ sampling points, $p (1+j) A /N_s$, with $p \in \{-N_s,\dots ,N_s\}$. Afterwards, we give a means to directly Fourier transform from the discretized N region to spatial-domain Gabor coefficients. We will only consider the northern (N) region of the complex spectral integration manifold since the E, W, and S region follow by analogy.

**Fig. 4:**
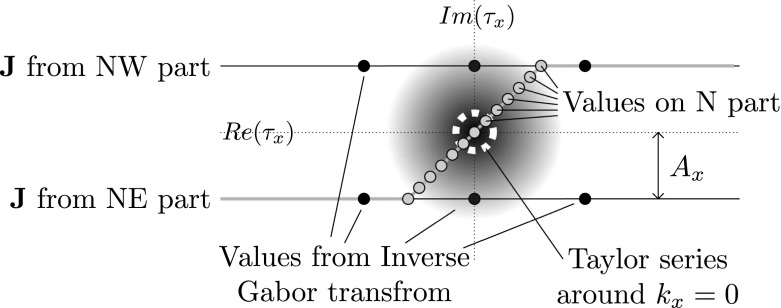
Illustration of the expansion for $N_\nu =1$ of the six known values (dark grey circles) from the NE and NW regions to values for the N region (light-gray circles)

For the calculation of the current density in the N region, function values of $\mathbf{J}^{\text {N}}$ are available at the lines $\tau _x = k_x \pm j A_x$, which were calculated via the Gabor representation in the NE and NW region. The analyticity of $\mathbf{J}$ allows to produce a Taylor expansion of $\mathbf{J}$ around $k_x=0$ from values at the lines $Im(\tau _x) = \pm A_x$. Afterwards, this Taylor expansion is used to calculate values of the contrast current density at the line $Re(\tau _x)=Im(\tau _x)$, where they are needed for discretization in the N region, as is shown in Fig. 4. Close to $k_x=0$, $\mathbf{J}^\text {N}$ can be approximated as

22 $$\begin{aligned} \mathbf{J}^\text {N}(k_x,k_y,z) \approx \sum _{n=0}^{4N_v+1} \frac{k_x^n}{n!} \mathbf{a}_n(k_y,z), \end{aligned}$$

where $\mathbf{a}_n(k_y,z) = \partial _{k_x}^n \mathbf{J}^\text {N}(k_x,k_y,z)$, and $4 N_v+2$ is the total number of terms in this Taylor expansion. Values for $\mathbf{J}^{\text {N}}(k_x \pm j A_x, k_y)$ can be obtained from the results for the NE and NW region, by using a fast Gabor transform Dilz and Beurden (2016), Bastiaans (1995) that yields values at $k_x =n \varDelta _{k_x} \pm j A_x$ for $n \in \mathbb {Z}$, with $\varDelta _{k_x}$ the spectral sample spacing corresponding to the Gabor frame. Values for $\mathbf{a}_n$ can be found by solving a small Vandermonde system (Press et al. 2007, Chapter 2.8). By constructing the vector $\underline{k}$ of $k_x$ values as $\underline{k} = (-N_v \varDelta _{k_x}-jA_x,\dots ,N_v \varDelta _{k_x}-jA_x,-N_v \varDelta _{k_x}+jA_x,\dots ,N_v \varDelta _{k_x}+jA_x)^T$, this Vandermonde system can be written as a matrix equation $\underline{\underline{K}} \cdot \underline{ \mathbf{a}}=\underline{\mathbf{j}}(k_y,z) = \mathbf{J}^{\text {N}}(\underline{k},k_y,z)$. The element $\underline{\underline{K}}_{mn}$ of the *m*’th row and *n*’th column of matrix $\underline{\underline{K}}$ is given by $\underline{\underline{K}}_{mn} = (\underline{k}_m)^n$, the *n*-th power of element *m* in $\underline{k}$. We solve this system using the inverse of $\underline{\underline{K}}$, i.e.

23 $$\begin{aligned} \underline{\mathbf{a}}(k_y,z) = \underline{\underline{K}}^{-1} \cdot \underline{\mathbf{j}}(k_y,z). \end{aligned}$$

Now that it is possible to express the Taylor coefficients $\mathbf{a}_n$ in terms of the $2 N_\nu +1$ samples on the NW-region, i.e. $\mathbf{J}(k_x-j A_x,k_y+j A_y,z)$, and the $2 N_\nu +1$ samples in the NE-region, i.e. $\mathbf{J}(k_x+j A_x,k_y+j A_y,z)$, they can be used to evaluate the Taylor expansion in Eq. (22) on the N-region, where $Im(\tau _x) =Re(\tau _x)$. We will write this as a matrix-vector product using the matrix $\underline{\underline{T}}$. The matrix $\underline{\underline{T}}$ transforms from a Taylor series to an equidistant sampling on the line $[-A_x-j A_x, A_x+j A_x]$. The elements are $\underline{\underline{T}}_{pm} = ( (1+j) p A_x/N_s)^m$, where $p \in \{-N_s, \dots ,N_s\}$ and where $m \in \{0, \dots , 4 N_\nu +1\}$, i.e.

24 $$\begin{aligned} \mathbf{J}^\text {N}_p(k_y,z)= [\underline{\underline{T}} \cdot \underline{\mathbf{a}}(k_y,z)]_p=[\underline{\underline{T}} \cdot \underline{\underline{K}}^{-1} \cdot \underline{\mathbf{j}}(k_y,z)]_p. \end{aligned}$$

We use the array of numbers $\mathbf{J}^\text {N}_{p,m_y,n_y,\ell }$ to represent the current in the N region of the complex integration domain. Index $p \in \{-N_s,N_s\}$ points to the set of piecewise-linear basis functions that are used in the $k_x$-direction on the line $\tau _x((1+j)p A/N_s)$. In the $k_y$-direction we use a Gabor frame, denoted here by indices $m_y$ and $n_y$, therefore the *y* dependence in Eq. (24) is replaced by this set of Gabor indices. Again, a set of $N_z$ PWL functions is used in the *z*-direction denoted by the index $\ell$.

Having dealt with the transformation to the N region, we will now deal with the transformation from the N region back to its spatial-domain counterpart. After multiplication of the contrast current density $\mathbf{J}^N_{p,m_y,n_y,\ell }$ with the Green function (see Sect. 3.1), the contribution of the North part of the scattered electric field yields $\mathbf{E}^{s,N}_{p,m_y,n_y,\ell }$. From this array we can make an approximation on the N region of the scattered electric field

25 $$\begin{aligned} \mathbf{E}^{s,\text {N}}(k_x + j k_x, k_y + j A_y, z)\approx \sum _{p=-N_s}^{N_s} \varLambda _{s_x,p}(k_x) \sum _{n_y,m_y}^{}g_{m_y,n_y}(k_y) \sum _{\ell =1}^{N_z} \varLambda _{z,\ell }(z) \mathbf{E}^{s,N}_{p,m_y,n_y,\ell }. \end{aligned}$$

Here $\varLambda _{s_x,p}$ are piecewise-linear (PWL) basis functions

26 $$\begin{aligned} \varLambda _{s_x,p}(k) = {\left\{ \begin{array}{ll} 1-\frac{|k - p \varDelta _{k_x}|}{ \varDelta _{k_x}} &{}\quad \text {if} \quad |k-p \varDelta _{k_x}|<\varDelta _{k_x} \\ 0 &{}\quad \text {if} \quad |k-p \varDelta _{k_x}|>\varDelta _{k_x} \end{array}\right. }, \end{aligned}$$

with width $\varDelta _{k_x} = A_x/N_s$ in the $k_x$-direction. To transform the N region of the scattered electric field $\mathbf{E}^{s,\text {N}}_{p,m_y,n_y,\ell }$ back to the spatial domain where it is discretized in the Gabor frame with coefficients $\mathbf{E}^{s,N}_{\mathbf{m},\mathbf{n},l}$, we use the Fourier transforms of the PWL functions in Eqs. (26) and (25), i.e.

27 $$\begin{aligned} I^{\text {N}}_p(x) = \int _{-A_x}^{A_x}dk_x \, \tau '(k_x) \, \varLambda _p(k_x) e^{j \tau _x(k_x) x }. \end{aligned}$$

Since the *x* direction is discretized using Gabor coefficients in the spatial domain, the *x*-dependence of this function $I^{\text {N}}_p(x)$ must be Gabor-transformed into Gabor coefficients $I^\text {N}_{p,m_x,n_x}$. These Gabor coefficients are calculated during initialization of the algorithm via e.g. Eq. (13) or a fast Gabor transform. Now the contribution of the N region to the scattered electric field in the spatial domain is given by

28 $$\begin{aligned} \mathbf{E}^{s}_{\mathbf{m},\mathbf{n},l} = \dots + \sum _{p=-N_s}^{N_s} \mathbf{E}^{s,N}_{p,m_y,n_y,\ell } I^N_{p,m_x,n_x}, \end{aligned}$$

where the dots indicate the contributions from the other eight regions to the scattered field.

Similar to regions of Type 1, some parts of the Green function are discretized using a continuation such as in Eq. (20), to avoid a branch cut. For example, for the N region the continuation is only needed in the *y*-direction, since a Gabor frame is employed in this direction only and a PWL discretization does not suffer from Gibbs ringing. The construction for a one-dimensional continuation is described in more detail in Dilz and Beurden (2017).

### Discretization in the region of type 3

For the middle (M) region, a two-dimensional version of the construction for the N region is used. Since the generalization is fairly straightforward, we will not write it down explicitly. The only difference here is that we use a total number of $2 N_m +1$ PWL functions per direction. We use a different number of PWL functions in this region since, depending on the simulation parameters, the accuracy can depend significantly on the choice for $N_m$. Since the middle part contains information of waves with small $\mathbf{k}_T$, it contains information about waves traveling almost parallel to the *z*-direction. Especially for scatterers that are larger in the *z*-direction, a larger $N_m$ is required.

An important remark on the use of Vandermonde matrices is that they are generally ill-conditioned when a uniform sampling is used, such as is the case in the NE and NW regions. In principle, this could lead to a poor conditioning of the $\underline{\underline{K}}$ matrix and therefore to an unstable inverse when the matrix is increased in size. However, the amount of information on the interval $\mathbf{k}_T \in [-A_x,A_x]\times [-A_y,A_y]$ is so small that large matrices are not needed.

There are two reasons that a relatively large number of PWL basis functions (typically $N_m>10$ and $N_s>10$) is needed in regions of Type 2 and 3. The first is that a PWL basis is relatively inefficient compared to a Gabor frame. For the second reason we have to look at both the spatial and the spectral domain. Since the contrast current density $\mathbf{J}$ is confined to a finite region only, its Fourier transform is fairly smooth. However, the scattered electric field $\mathbf{E}^s$ is not confined to the simulation domain, and therefore its Fourier transform is much less smooth. On the Type-1 and Type-2 regions this lack of smoothness is compensated by a representation on complex spectral coordinates $\tau$, where the Green function is much smoother. However, the Type-3 region is not shifted as far into the complex plane as the Type 1 and Type 2 regions, and therefore the Green function is less smooth in this region. Since the Green function is implemented recursively, for intermediate results, i.e. the scattered field in between $z_{min}$ and $z_{max}$, this lack of smoothness should be represented accurately. Afterwards, when the transformation to the spatial domain is performed, this roughness on the M region corresponds to contributions outside the simulation domain, but ignoring the roughness is not an option since it leads to accumulating errors in the recursive handling of the Green function. This is especially important when $z_{max}-z_{min}$ is large compared to the wavelength.

### Correspondence between simulation parameters and accuracy

Since there are many simulation parameters, it is not trivial to find values for these parameters that produce both a good accuracy and short computation time. This list is intended to clarify which simulation parameters influence which part of the algorithm. This list is intended as a general guideline for optimal results.

1. Start with a Gabor frame with $X = Y = \lambda$, the wavelength of the light-source, and $\alpha = \beta = \sqrt{2/3}$.
2. Choose $m_x > 3 + W_x / \alpha X$ and similarly $m_y > 3 + W_y /\alpha Y$. Choose $n_x = n_y > 5 \max _{x \in {\mathscr {D}}}(1+\chi (x))$, which guarantees at least 11 unknowns per wavelength per direction. Test whether a function (e.g. a Gaussian with width *X*), can be represented with the required accuracy over the entire simulation domain ${\mathscr {D}}$. When the accuracy is too low everywhere, increase $\mathbf{n}$, when the accuracy is too low at the boundary of ${\mathscr {D}}$ only, increase $\mathbf{m}$.
3. Start at $A_x = 3/W_x$ and $A_y=3/W_y$, $N_v= 1$, $N_s = 10$ and $N_m = 10$. Test whether a set of spatially and spectrally localized functions (e.g. modulated Gaussians that are shifted along the entire simulation domain) can be transformed to the complex spectral integration manifold and back again with the required accuracy. Note that the exponential function in Eq. (17) reduces the accuracy of the Gabor frame. Therefore, a simultaneous decrease of $A_x$ and increase of $\mathbf{m}$ improves the accuracy in the transformation between the spatial and spectral domain. Especially when a high accuracy is needed, $N_\nu$ might need to be increased when the error in the N, E, S, W and M regions is too large. Also an increase in $N_s$ and $N_m$ can be considered when the error in the PWL interpolation is found to be too large.
4. The Green function (Eq. 8) contains a factor $\gamma ^{-1}$, that has a strongly peaked behavior around $|\mathbf{k}_T| = \sqrt{\varepsilon _{rb,i}} k_0$. Test whether the function $\gamma ^{-1}$ is represented accurately enough by the Gabor frame with the current parameters. Otherwise $A_x$ can be increased (which decreases the accuracy in the previous step) or $\mathbf{m}$ can be increased (which increases the computation time, but leaves the accuracy in the previous step invariant).
5. Especially for large $z_{max} - z_{min}$, a lot of information is stored in the M region, which contains information about waves traveling in a narrow cone around the *z* axis. The main culprit here is the function $e^{- \gamma (z_{max}-z_{min})}$ in Eq. (8). Choose $N_m$ such that this function can be well approximated in the M region.
6. Another simulation can be run with lower $\mathbf{n}$ values. When both results agree well, convergence in the spectral range $\mathbf{n}$ has been reached, otherwise $\mathbf{n}$ should be increased for higher accuracy.

## Efficient field-material interaction

Formulating the field-material interaction as proposed in Eq. (4) yields poor convergence since it violates the Li-rules (1996). We propose to use a normal-vector field approach (Popov and Nevire 2001; Beurden and Setija 2017). In Dilz et al. (2017) it is shown that when the Li-rules are satisfied, good convergence is reached in a continuous spectral discretization in a formulation similar to the RCWA formulation by Granet and Guizal (1996). We follow the same approach as Popov and Nevire (2001), Beurden (2011, 2012), Beurden and Setija (2017), van Beurden (2013) in constructing normal-vector fields and the following is intended as a short summary of that method.

When the permittivity is discontinuous at a material interface, it is observed that the electric field $\mathbf{E}$ normal to the surface is discontinuous, but the electric flux density $\mathbf{D}$ normal to such a surface is continuous. Therefore, in the field-material interaction in Eq. (4), both $\chi$ and the normal component of $\mathbf{E}$ are discontinuous and multiplication of two discontinuous functions represented by Gabor shows poor convergence (Dilz et al. 2017), since it violates the Li-rules (1996). An auxiliary field $\mathbf{F}$ is introduced that is composed of $\mathbf{D}$ in the direction normal to every surface of discontinuous $\chi$ and $\mathbf{E}$ parallel to each of those surfaces. Since this fixes the choice of $\mathbf{F}$ only at the boundaries of dielectric objects, there is much freedom in choosing it away from the interfaces. Normal-vector fields (Popov and Nevire 2001; Rafler et al. 2008; Götz et al. 2008; Beurden and Setija 2017) can be a good tool to systematically construct an auxiliary field $\mathbf{F}$.

Since we use Gabor coefficients only in the transverse plane, we apply the normal-vector field formulation only in the transverse plane. For objects with interfaces that are not aligned with the *z* or transverse plane, a staircasing approximation is needed. When $\mathbf{N}_T(\mathbf{x})$ is a vector field of unit amplitude that is directed normal to the transverse part of all discontinuous surfaces in $\chi$ and when $\alpha (\mathbf{x})$ is a scalar function that equals one at these discontinuities, these functions can be used to construct the desired auxiliary field $\mathbf{F}$ as

29 $$\begin{aligned} \mathbf{F}(\mathbf{x}) =\mathbf{E}(\mathbf{x}) + \mathbf{N}_T(\mathbf{x}) \left[ \left( \frac{\alpha (\mathbf{x})}{\varepsilon _0 \varepsilon _{rb,i}} \mathbf{D}(\mathbf{x}) - \mathbf{E}(\mathbf{x}) \right) \cdot \mathbf{N}_T(\mathbf{x}) \right] . \end{aligned}$$

The field-material interaction in Eq. (4) can be re-written as

30 $$\begin{aligned} \mathbf{J}(\mathbf{x}_T,z) = \, [\chi C_\varepsilon ](\mathbf{x}_T,z) \mathbf{F}(\mathbf{x}_T,z), \end{aligned}$$

and the electric field can be recovered from

31 $$\begin{aligned} \mathbf{E}(\mathbf{x}_T,z) = [C_\varepsilon ](\mathbf{x}_T,z) \mathbf{F}(\mathbf{x}_T,z), \end{aligned}$$

where the Cartesian component *i* of the electric field due to the Cartesian component *j* of auxiliary field *F* is calculated by employing operator $C_\varepsilon$ defined as

32 $$\begin{aligned} {[}C_{\varepsilon }(\mathbf{x})]_{ij} = \delta _{ij}+\mathbf{N}_{T,i}(\mathbf{x})\mathbf{N}_{T,j}(\mathbf{x}) \left[ \frac{1}{\alpha (\mathbf{x}) (1+\chi (\mathbf{x}))} - 1 \right] \end{aligned}$$

with $\delta _{ij}$ denoting the Kronecker delta and similarly

33 $$\begin{aligned} {[}\,\chi C_{\varepsilon }(\mathbf{x})]_{ij} = \chi (\mathbf{x}) {[}C_{\varepsilon }(\mathbf{x})]_{ij}. \end{aligned}$$

Using this normal-vector field approach, there is a great freedom for the shape of the scatterer. Depending on the scatterer shape, a suitable $\mathbf{N}_T$ and $\alpha$ must be chosen. It is possible to choose $\mathbf{N}_T$ and $\alpha$ for basic geometric elements, such as rectangular blocks, triangular prisms, or circular cylinders. More intricate objects can then be constructed from combinations of a variety of such objects. More details and examples of this construction can be found in Beurden and Setija (2017).

## Numerical results

**Fig. 5:**
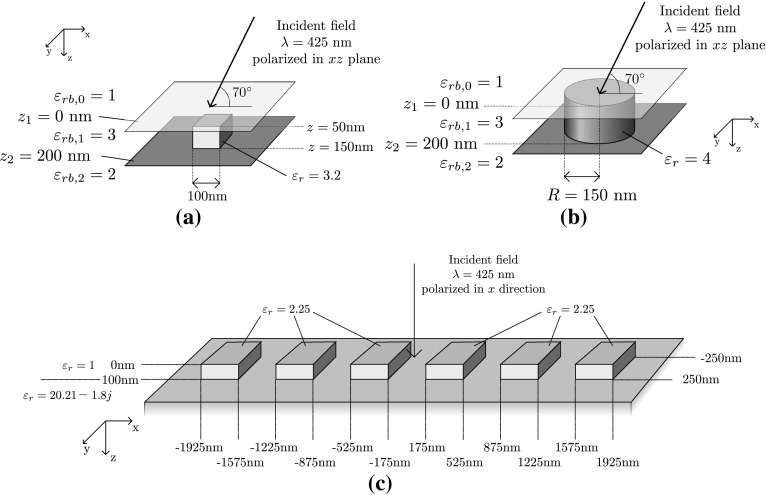
**a** The scattering setup for a small, low-contrast 100 nm cube embedded in a multilayered medium. **b** A cylinder, embedded in the same multilayered medium. **c** A finite grating consisting of six repeating blocks located on top of a substrate

We have chosen three testcases to validate the present algorithm. As a reference to validate our results we use the commercial FEM code JCMWave (Burger et al. 2013). Our goal is to achieve an accuracy of $10^{-3}$, which is sufficient in our applications, e.g. due to measurement noise or fabrication tolerances. To achieve high accuracy in the validation, we use a relatively small, low-contrast scatterer in the first testcase. A small dielectric cube is embedded in a dielectric medium as shown in Fig. 5a, together with the remaining details of the setup. The incident wave is characterized by a Cartesian wave vector with components $\mathbf{k} = (-k_0 \sin (70^\circ ) , 0 ,k_0 \cos ( 70^\circ ))$, with the electric field polarized in the *xz*-plane and with unit amplitude.

We choose a Gabor frame with $X=Y=80$ nm, $\alpha = \beta = \sqrt{2/3}$. For the highest accuracy we use a Gabor frame, Eq. (12), restricted to $m_x,m_y \in \{-7,\dots ,7 \}$ and $n_x,n_y \in \{-10,\dots ,10\}$, which equals one basis function per 3.1 nm. In the *z*-direction we use a step size of 2.5 nm. For the sampling of the regions of Type 2 and 3 we use $N_\nu = 2$, $N_s=N_m=15$.

**Fig. 6:**
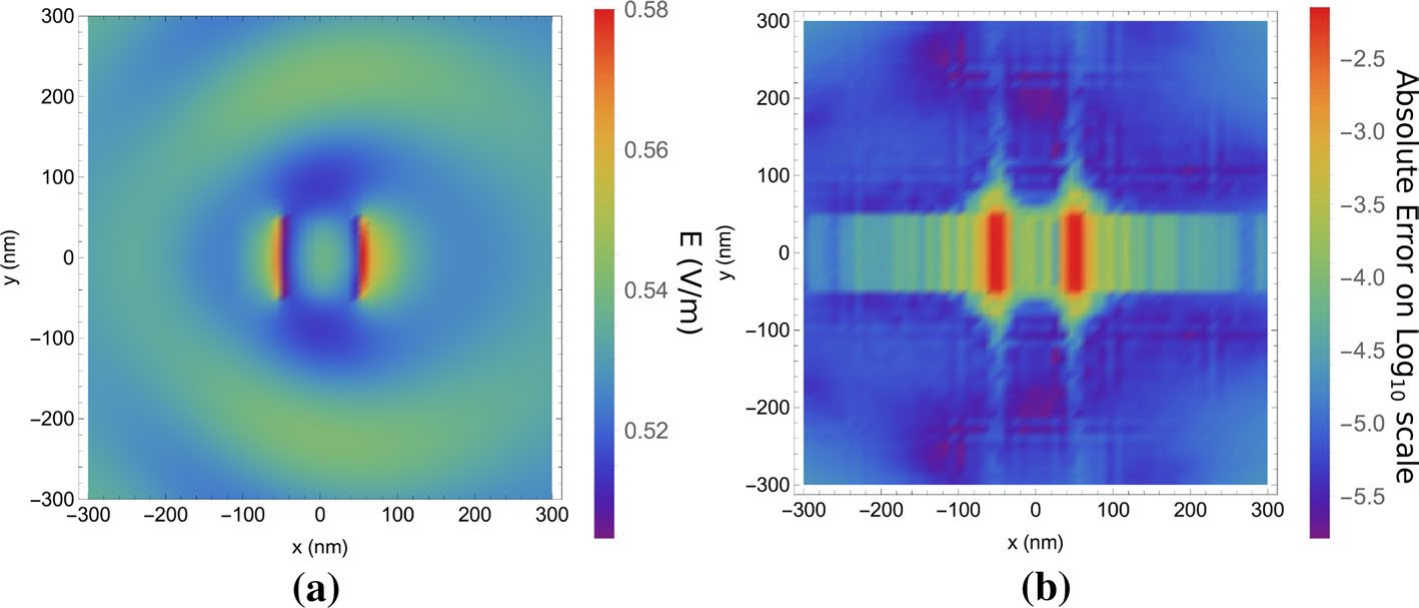
The electric field at the $z=100$ nm plane for the scattering case in Fig. 5a. In Figure **a** $|\mathbf{E}|$ is plotted for an incident plane wave with unit amplitude and in Figure **b** this is compared to the results obtained using JCMWave

With these simulation parameters, the simulation domain in the *xy*-plane extends over a larger region than the scatterer itself, as is visible in Fig. 6a. In this figure, the norm of $\mathbf{E}$ is shown on the plane $z=100$ nm. In Fig. 6b the absolute difference between results from the present algorithm and the JCMWave reference are shown. Over large regions of the simulation domain the absolute difference is smaller than $10^{-5}$. From $y=-50$ nm to $y=50$ nm and close to the edges of the cube agreement between both simulations is not as good as on the rest of the simulation domain. This is caused by Gibbs-ringing at the discontinuities in the electric field, especially in the *x*-component. The field-material interaction operators $C_\varepsilon$ and $\varepsilon C_\varepsilon$ of Sect. 5 exhibit this Gibbs ringing because they are truncated in the spectral domain. This Gibbs ringing is therefore also present in the scattered electric field.

**Fig. 7:**
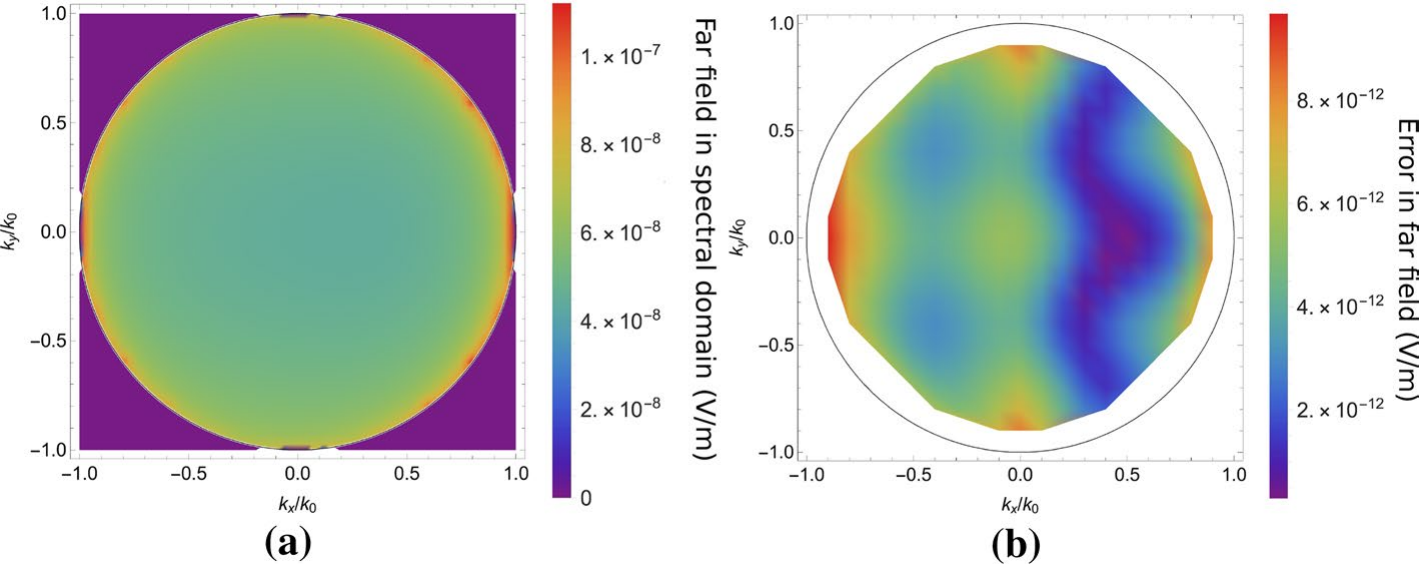
The far field for the case in Fig. 5a as a function of the transverse wavenumber $\mathbf{k}_T/k_0$, scattered back into the half-space $z<0$. In **a** the modulus $|\mathbf{E}^s|$ of the scattered electric field is shown. In **b** the difference between a JCMWave validation run and the present algorithm is shown. An average relative error of $4 \times 10^{-5}$ was observed. Since an interpolation of the reference data is used that is not accurate at the edge of the radiation circle, the far field data is truncated for large $\mathbf{k}_T$

Since this Gibbs ringing has a very small spatial period, it does not radiate into the far field. Because the far field is the most interesting for the application, we use the far field as a reference for the accuracy of the method. As can be observed in Fig. 7, the error in the far field is much lower than in the near field, because the absence of the Gibbs ringing. The average relative difference with an $\mathscr {L}^2$-norm in the far field data equals $4 \times 10^{-5}$. Clearly, the far-field results agree much better than the near-field results. The small size of the scatterer and its low contrast results in a far field pattern that does not vary much with the angle. This example is therefore somewhat uninteresting, however, it has the advantage that the FEM reference could achieve a high accuracy in a multilayered scattering problem.

**Fig. 8:**
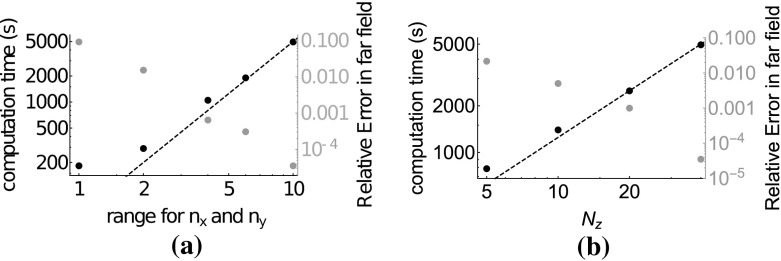
In Figure **a** both the computation time and the relative error in the far field, computed as the average of Fig. 7b for a range of $n_x$ and $n_y$ for the Gabor frame. In **b** the same is shown, but now for different sampling $\varDelta _z$ in the *z*-direction

In Fig. 8, we show how both the accuracy and the computation time scale with the number of unknowns used in the calculation. The horizontal axis in Fig. 8a contains the sample density, which was lowered by decreasing the range of the $\mathbf{n}$-index in Eq. (12), where $n_x,n_y \in \{-r,\dots ,r\}$ from $r=10$ down to $r=1$. This corresponds to a sample density $1/\varDelta _x = 1/\varDelta _y = (2 r+1) / \sqrt{\alpha /\beta } X$. The other simulation parameters were kept constant throughout this sweep. The results suggest that the computation time scales as $O(1/\varDelta _x \varDelta _y) = O(N_x N_y)$. Since FFTs are used, we expect an $O(N_x N_y \log (N_x N_y))$ behaviour, but the logarithms are apparently negligible compared to other parts of the algorithm at this simulation size. Fig. 8b shows a clear $O(N_z)$ behaviour, which is expected from Gohberg and Koltracht’s recursion (1985).

**Fig. 9:**
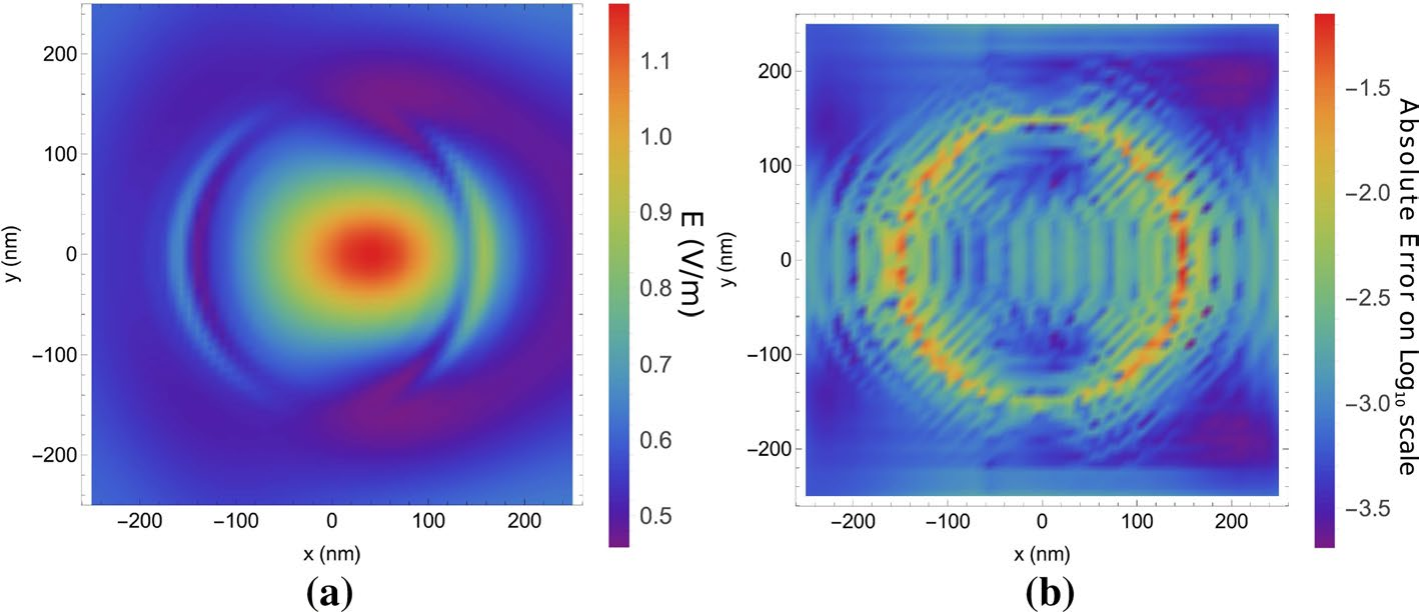
The electric field in the plane $z=10$ nm plane for the scattering case in Fig. 5b. In Figure **a** $|\mathbf{E}|$ is plotted for an incident plane wave with unit amplitude and in Figure **b** this is compared with the results obtained using JCMWave

The second example for which we provide computational data consists of a dielectric cylinder embedded in a multilayered medium as is described in Fig. 5b. In Fig. 9, the electric field is shown at $z=10$ nm for $X = Y = 100$ nm, $\alpha = \beta = \sqrt{2/3}$ and $m_x,m_y \in \{-4,\dots ,4 \}$ and $n_x,n_y \in \{-7,\dots ,7\}$, which equals one basis function per 5.1 nm. In the *z*-direction, 41 basis functions are used with stepsize $\varDelta _z=2.5$ nm. For the sampling of the regions of Type 2 and 3 we use $N_\nu = 2$, $N_s=N_m=15$. From Fig. 9b it is clear that the difference in the result from the present algorithm compared to the JCMWave reference is somewhat larger than for the previous case. However, this is due to a less accurate reference that was calculated with a lower order *p*-refinement. A higher order *p*-refinement was not feasible with the available computational resources.

**Fig. 10:**
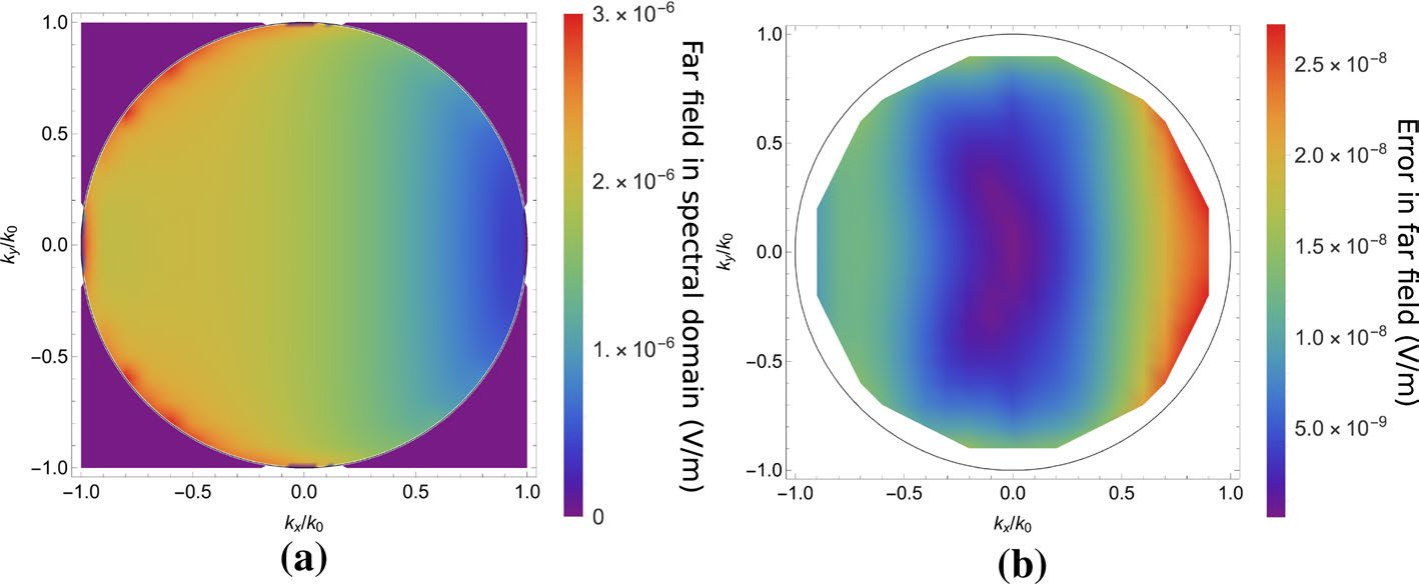
The far field for the case in Fig. 5b as a function of the transverse wavenumber $\mathbf{k}_T/k_0$, scattered back into the half-space $z<0$. In **a** the modulus $|\mathbf{E}^s|$ of the scattered electric field is shown. In **b** the difference between a JCMWave validation run and the present algorithm is shown. An average relative error of $2.8 \times 10^{-3}$ was observed

In Fig. 10, the absolute value of the far field reflected into the upper half-space is plotted. The relative error for the simulation in the far field is $2.8 \times 10^{-3}$, which is significantly larger than for the cubic scatterer, because of the reference, with lower accuracy.

We have calculated the far field with a finer discretization, to show the convergence of the algorithm. We emphasize that the present algorithm does not excel at small computational domains. However, for a small computational domain a more accurate validation result was feasible than for a very large computational domain. The reason that the present algorithm is relatively slow for small simulation domains is that a lower limit on the number of unknowns in the *x* and *y*-direction exist, since the Gabor frame (as we choose it) is inaccurate over a range of at least three window widths $\alpha X$ (see Eq. (12)) distance from the point at which the Gabor frame is truncated to zero. Therefore, at least seven windows are needed for an accuracy of $10^{-3}$ in the middle of the computational domain, both for the spatial index $m_x$ as well as for the spectral index $n_x$ in Eq. (12). Consequently, at least seven values for index $m_x$ and seven values for index $n_x$ are needed, which amounts to a total of 49 coefficients per direction at minimum. Since we use a Gabor frame in two dimensions, a minimum of 2401 unknowns is needed for a minimum simulation domain. Note that we assumed the use of a Gabor frame with $\alpha = \beta = \sqrt{2/3}$ and the Moore-Penrose inverse to calculate the dual window, where the accuracy increases exponentially with the distance to the truncated coefficients for this choice (Böcskei and Janssen 2003) for sufficiently smooth functions. This effect only exists for small simulation domains. For large simulation domains, the number of unknowns at the edge of the simulation domain is negligible.

**Fig. 11:**
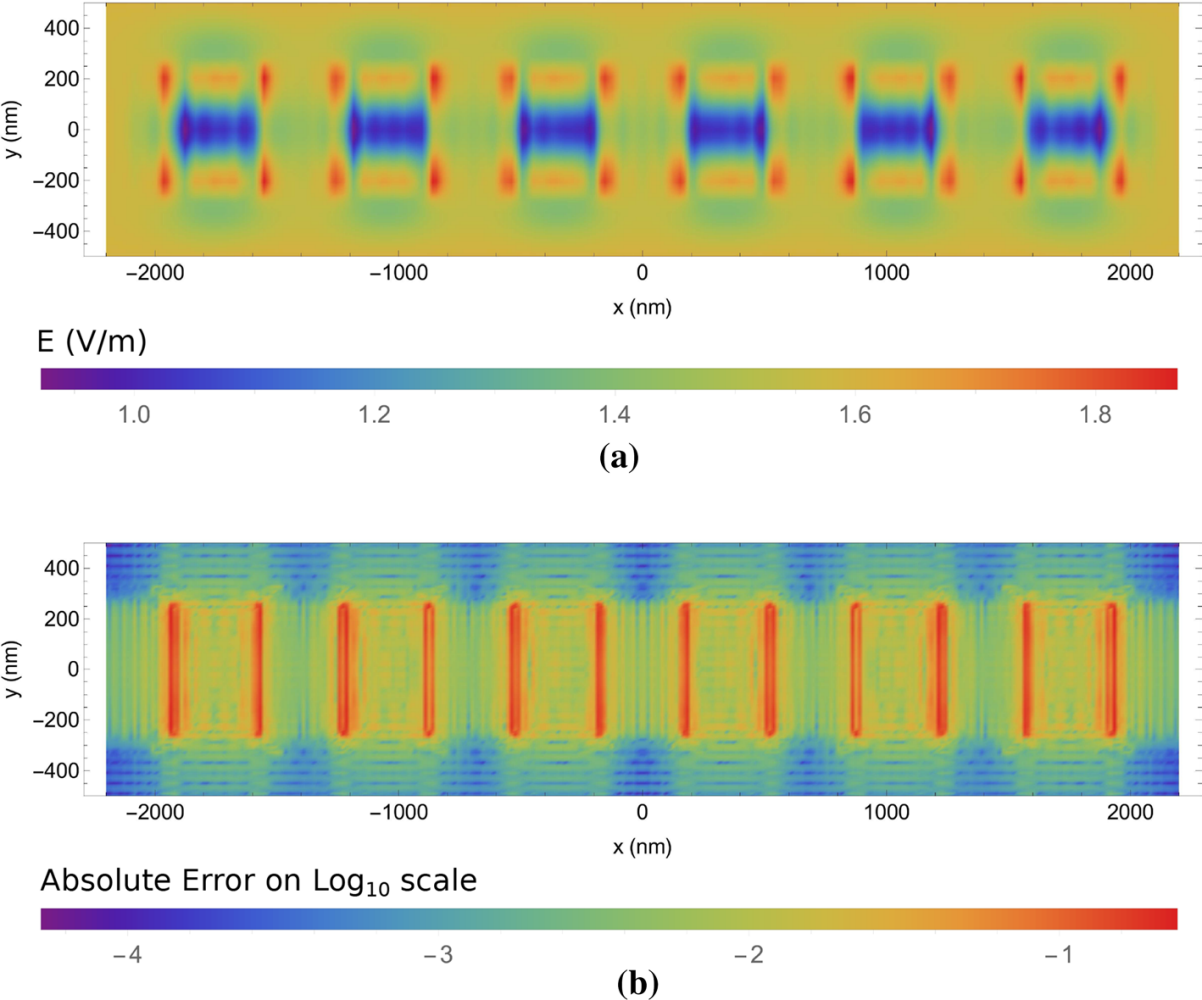
The electric field in the plane $z=10$ nm plane for the scattering setup in Fig. 5c. In Figure **a** $|\mathbf{E}|$ is plotted for a normally incident plane wave with unit amplitude and in Figure **b** this is compared with the results obtained using JCMWave

**Fig. 12:**
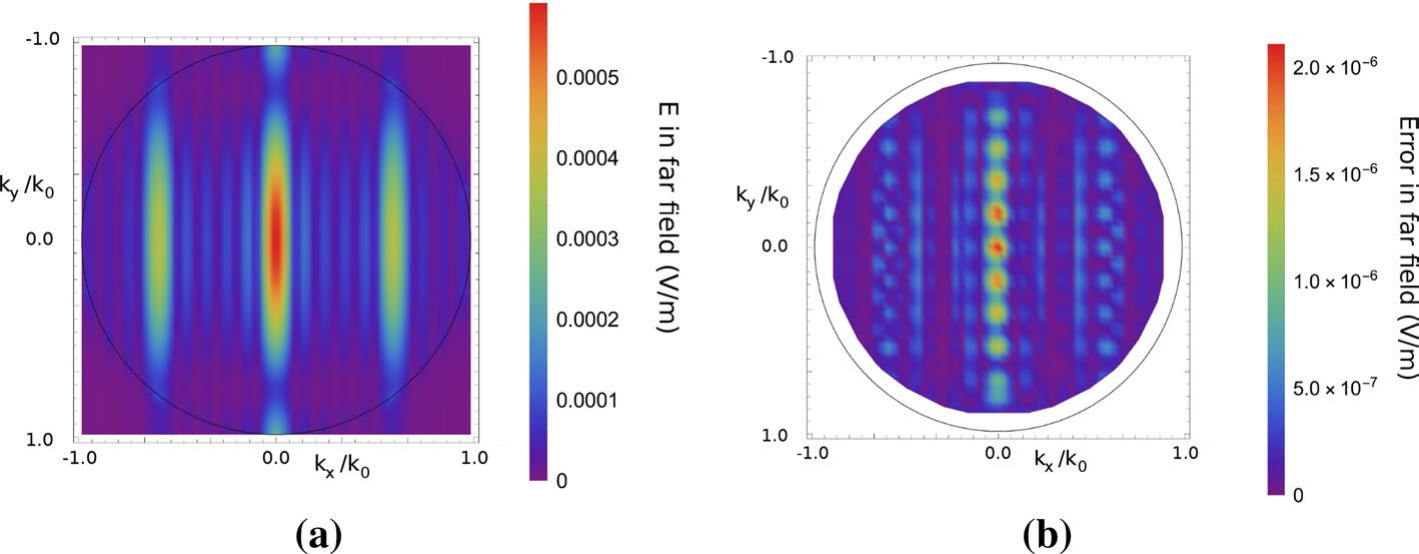
The far field for the case in Fig. 5c as a function of the transverse wavenumber $\mathbf{k}_T/k_0$, scattered back into the half-space $z<0$. In **a** the modulus $|\mathbf{E}^s|$ of the scattered electric field is shown. In **b** the difference between a JCMWave validation run and the present algorithm is shown. An average relative error of $2.5 \times 10^{-3}$ was observed

The third and final example for which we computed the scattered electric field consists of six dielectric blocks of $350\times 500\times 100$ nm deposited on a slightly lossy dielectric substrate as is shown in Fig. 5c. In Fig. 11, the electric field is shown at $z=10$ nm for $X = Y = 500$ nm, $\alpha = \beta = \sqrt{2/3}$ and $m_x \in \{-7,\dots ,7 \}$, $m_y \in \{-4,\dots ,4 \}$ and $n_x,n_y \in \{-7,\dots ,7\}$, which equals one basis function per 27 nm. In the *z*-direction, 21 basis functions are used with stepsize $\varDelta _z=5$ nm. For the sampling of the regions of Type 2 and 3 we use $N_\nu = 2$, $N_s=N_m=20$. Since the scatterer is larger than in the previous examples, it was efficient to choose larger window widths *X* and *Y*, which results a coarser sampling. From Fig. 11b it is clear that this coarser sampling generates a somewhat more pronounced Gibbs-ringing from the edges. However, in the far field, which is shown in Fig. 12, the average relative difference with the JCMWave reference calculation is similar to that for the cylinder case, i.e. $2.5 \times 10^{-3}$, where the estimated relative accuracy of the reference calculation was on the order $2\times 10^{-3}$. Even though the scatterer extends much wider in the *xy* plane, the number of unknonws in the *xy*-plane was increased by only a factor 5 / 3, while the accuracy in the far field remained similar. This clearly shows that the present method performs better for scatterers larger than a wavelength in size.

## Conclusion

A volume integral equation for 3D scattering from finite dielectric objects embedded in a dielectric layered medium was presented in the mixed spatial and spectral domain and an algorithm based on Gabor frames was presented for the discretization. The algorithm employs a mixed spatial spectral formulation and Gabor frames for the discretization. A representation of the Green function, contrast current density, and scattered electric field on a complex integration manifold is employed in the spectral domain. A normal-vector field formulation in the transverse spatial domain is employed to improve the convergence in the field-material interaction.

The accuracy of the present algorithm was compared to a FEM algorithm. The results of both algorithms in the far field agree with each other up to the $4\times 10^{-5}$ in one small numerical example. In the other two examples an agreement up to $2.8 \times 10^{-3}$ and $2.5 \times 10^{-3}$ were observed, because the FEM algorithm did not fully converge with the computational resources at hand. Numerical evidence was presented that the computational complexity of the present algorithm scales as $O(N \log N)$ with the number of unknowns.
